# Ultrasound measurements of croup fat thickness and liver echogenicity as indicators for lipomobilization in donkeys (*Equus Africanus asinus*) with fasting-induced hyperlipidemia

**DOI:** 10.1186/s12917-025-04999-z

**Published:** 2025-09-09

**Authors:** Abeer Abd El-Wares Mahmoud, Hanan Kamal Elsayed, Hussein Awad Hussein

**Affiliations:** 1 Veterinary Internal Medicine, Department of Animal Medicine, Faculty of Veterinary Medicine, Assiut University, Assiut, 71526 Egypt; 2 Department of Animal Medicine and Infectious Diseases, School of Veterinary Medicine, Badr University in Assiut, Assiut, 19952 Egypt

**Keywords:** Croup fat thickness, Equid, Fasting, Hyperlipidemia, Liver echogenicity, Triglycerides

## Abstract

**Background:**

Disturbances in lipid metabolism are usually associated with hyperlipidemia, which is commonly observed in donkeys with inappetence or anorexia. The diagnostic utility of ultrasound measurements of croup fat thickness (CFT) and relative liver echogenicity for lipomobilization in donkeys with fasting-induced hyperlipidemia was investigated. A prospective observational control study involving 25 donkeys was conducted, and the animals were randomly assigned to a fasting group (FG, *n* = 20) and a control group (CG, *n* = 5). In the FG, the experiment period (10 days) consisted of two stages, the fasting stage (4 days) and the post-fasting stage (6 days).

**Results:**

On six occasions, the following were evaluated: body weight (BW), body condition score (BCS), ultrasound subcutaneous CFT, ultrasound gluteal muscle thickness, liver ultrasonography, and blood metabolites. The ultrasound CFT was significantly reduced at 4 days of fasting (*P* < 0.05). Donkeys with ≥ 7 mm of CFT before fasting were 6 times more likely to develop hyperlipidemia post-fasting (*P* < 0.01). Hepatic ultrasonography showed no hepatomegaly. Decrements of the portal vein (PV) diameter were noticed during fasting. The hepatic relative echogenicity (RE) significantly increased after 4 days of fasting, and then decreased after fasting (*P* < 0.05). The RE of ≥ 78 is a critical threshold for diagnosing hyperlipidemia (*P* < 0.001). The serum concentrations of triglycerides, total cholesterol, very low-density lipoproteins, high-density lipoproteins, and low-density lipoproteins peaked at 4 days of fasting (*P* < 0.05). The serum concentrations of FFA increased during fasting (*P* < 0.05) and then dropped after fasting.

**Conclusion:**

Fasting-induced hyperlipidemia is associated with reductions in CFT with concurrent increased RE, suggesting lipomobilization. CFT and RE could be used as diagnostic tools for hyperlipidemia. Reversible variations in serum metabolites could be noticed in donkeys as complications of fasting-induced hyperlipidemia; therefore, therapy may be unnecessary especially in less severe cases.

## Background

Hyperlipidemia is a lipid metabolism disorder resulting in the accumulation of blood triglycerides (TGs) in equine. It may develop as a physiological response to the negative energy balance resulting from inappetence or anorexia [1]. Exposure to a negative energy balance leads to lipolysis, with the subsequent release of free fatty acids (FFAs) into the liver, where they are re-esterified into triglycerides (TG) and then re-released into the bloodstream, leading to variable degrees of hyperlipidemias [2]. Donkeys are more susceptible to hyperlipidemia because they can coexist with poor-quality rations, stress during transportation, and fasting [3, 4]. Moreover, the onset of hyperlipidemia is not solely due to this factor but is also related to diverse intestinal microbial communities that may play a critical role in nutritional metabolism, feed efficiency, and the health of donkeys [5].

Body condition is a main criterion of animal welfare and a useful indicator of body fat reserve, with subsequent assessment of donkey nutritional status and well-being [6]. There are various methods used for evaluating body condition in donkeys, including animal-based morphometric indices such as body condition score (BCS) and body weight [7] and measurement of subcutaneous fat via ultrasound [8]. The liver is the key organ for the metabolism of mobilized fats in donkeys suffering from negative energy balance [2]. Excessive fat mobilization may overwhelm the liver’s capacity, leading to TG accumulation and hepatic lipidosis [9]. Because of its accessibility and noninvasiveness, safety, and ease of use, liver ultrasonography has become the method of choice in the diagnosis of hepatic lesions in horses [10] and donkeys [11].

As a laboratory diagnostic tool, blood metabolites reflecting fat and carbohydrate metabolisms should be measured to assess the homeostatic body response to fasting-induced hyperlipidemia. Many studies have reported the effect of fasting or feed restriction on blood biochemical indices in horses and donkeys [12, 13]; however, no reports stated the post-fasting variations in these metabolites. Moreover, to the best of the authors’ knowledge, no reports have evaluated the effect of fasting on ultrasound measurements of subcutaneous fat and gluteal muscle thickness or on hepatic ultrasonography in donkeys. Therefore, the objective of this study was to evaluate the use of ultrasound measurements of croup fat thickness (CFT) and liver relative echogenicity (RE) as diagnostic tools for lipomobilization in donkeys with fasting-induced hyperlipidemia

## Results

### Effect of fasting-induced hyperlipidemia on physical parameters and body condition

**Fig. 1:**
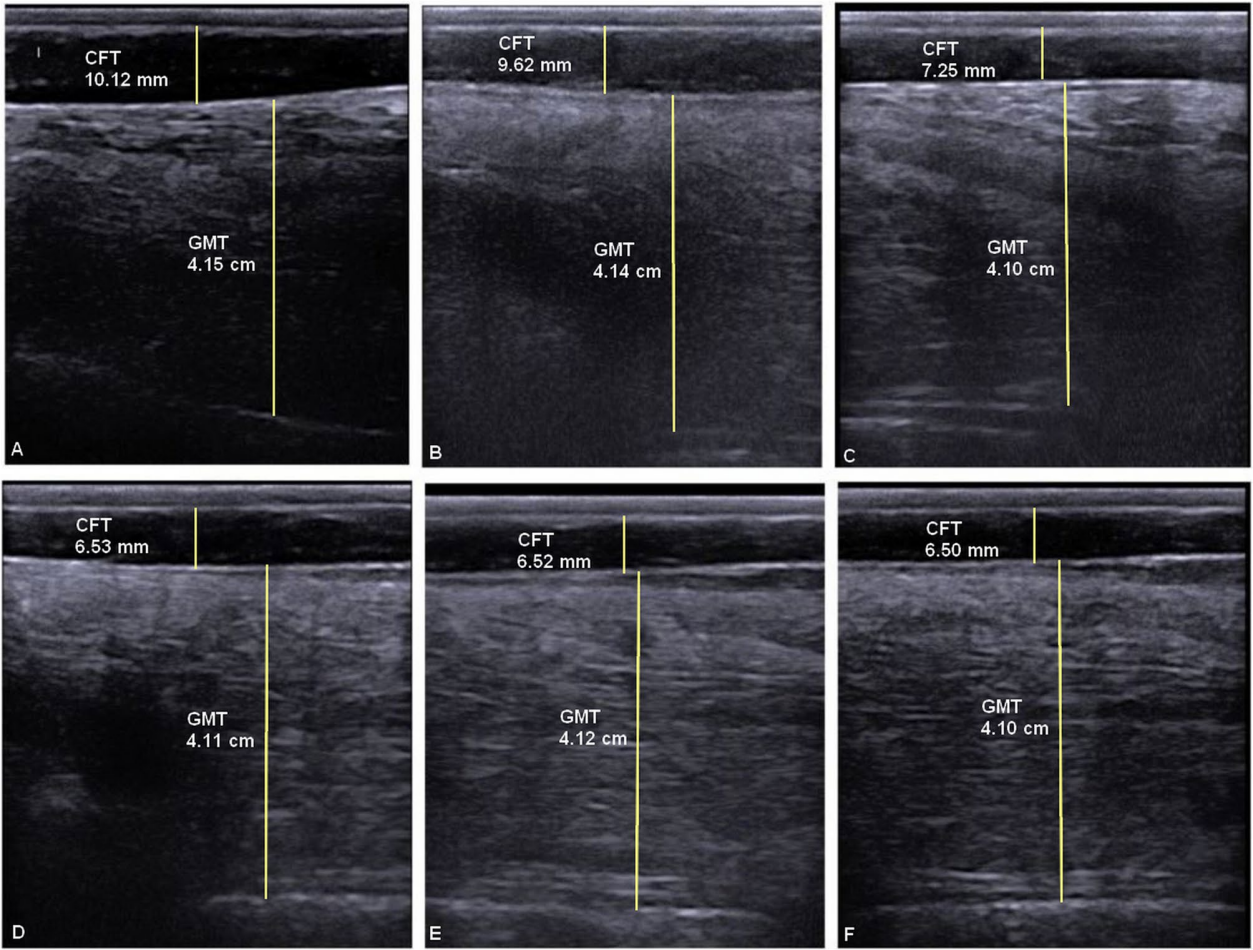
Ultrasound images show the variations in ultrasound measurements of croup fat (CFT) and gluteal muscle thicknesses (GMT) for a donkey in the fasting group throughout the study, just before fasting (A), after 2 and 4 days of fasting (B and C, respectively), and after 5, 7, and 10 days of the study (D, E and F, respectively)

Generally, compared to control animals, donkeys showed mild degrees of dullness and depression during the fasting stage, which were managed through avoiding nagging and improving the donkeys’ environment and social interactions. Table 1 summarizes the comparative evaluation of the body condition methods used in the fasting and control groups. The mean BW (*P* < 0.01) and ultrasound CFTs (*P* < 0.05) revealed significant differences between the control and fasting groups, although no variations were noticed between both groups at the baseline. Fasting time had no considerable effect on BW, BCS, and ultrasound-related GMT (*P* > 0.05), except for ultrasound CFT (*P* < 0.001), for which there was a significant group/time interaction (*P* < 0.001), indicating that ultrasound-related CFTs behaved differently over time between groups. In the fasting group, the ultrasound CFT decreased gradually after fasting till the end of the study (*P* < 0.05) (Fig. 1).

**Table 1:** The effect of fasting-induced hyperlipidemia on the body condition of donkeys (*Equus asinus*)

Parameters	Groups	Days relative to fasting^†^						Repeat measures analysis of variance^‡^		
			Fasting stage		Post-fasting stage					
	(Number)	0	2	4	5	7	10	Group	Time	Group × time
BW (Kg)	FG (*n* = 20)	191 ± 3.8	191 ± 2.4^*^	190 ± 2.3^*^	190 ± 2.7^*^	190 ± 2.5^*^	191 ± 1.2^*^	0.01	0.955	0.853
	CG (*n* = 5)	184 ± 3.4	182 ± 2.1	181 ± 2.2	182 ± 2.8	180 ± 1.9	180 ± 2.7			
BCS (unit)	FG (*n* = 20)	2.7 ± 0.3	2.8 ± 0.1	2.6 ± 0.3	2.5 ± 0.3	2.5 ± 0.1	2.5 ± 0.1	0.244	0.357	0.709
	CG (*n* = 5)	2.3 ± 0.3	2.1 ± 0.4	2.0 ± 0.6	2.2 ± 0.3	2.1 ± 0.2	2.1 ± 0.3			
Ultrasound CFT (mm)	FG (*n* = 20)	8.3 ± 1.3^a^	8.0 ± 0.3^a*^	7.3 ± 0.4^b*^	7.5 ± 0.2^b*^	7.6 ± 0.2^b*^	8.0 ± 0.3^b*^	0.05	0.000	0.000
	CG (*n* = 5)	6.8 ± 1.2	6.5 ± 0.4	6.3 ± 0.2	6.4 ± 0.7	6.5 ± 0.6	6.6 ± 0.6			
Ultrasound GMT (cm)	FG (*n* = 20)	3.8 ± 0.3	3.7 ± 0.5	3.7 ± 0.3	3.6 ± 0.5	3.6 ± 0.2	3.6 ± 0.3	0.055	0.875	0.588
	CG (*n* = 5)	3.3 ± 0.3	3.3 ± 0.4	3.2 ± 0.1	3.2 ± 0.1	3.3 ± 0.3	3.2 ± 0.1			

*Abbreviations*: *BW* body weight, *BCS* body condition score, *CFT* Croup fat thickness, *GMT* gluteal muscle thickness, *FG* fasting group, *CG* control group

^†^ Values are mean ± standard error of the mean

^‡^ F-values of groups, times relative to fasting, and group–time interactions

^ªᵇ^Means in the same row differ significantly (*p* < 0.05)

^*^Means in the column of the same parameter differ significantly between control and fasting groups (*p* < 0.05)

**Fig. 2:**
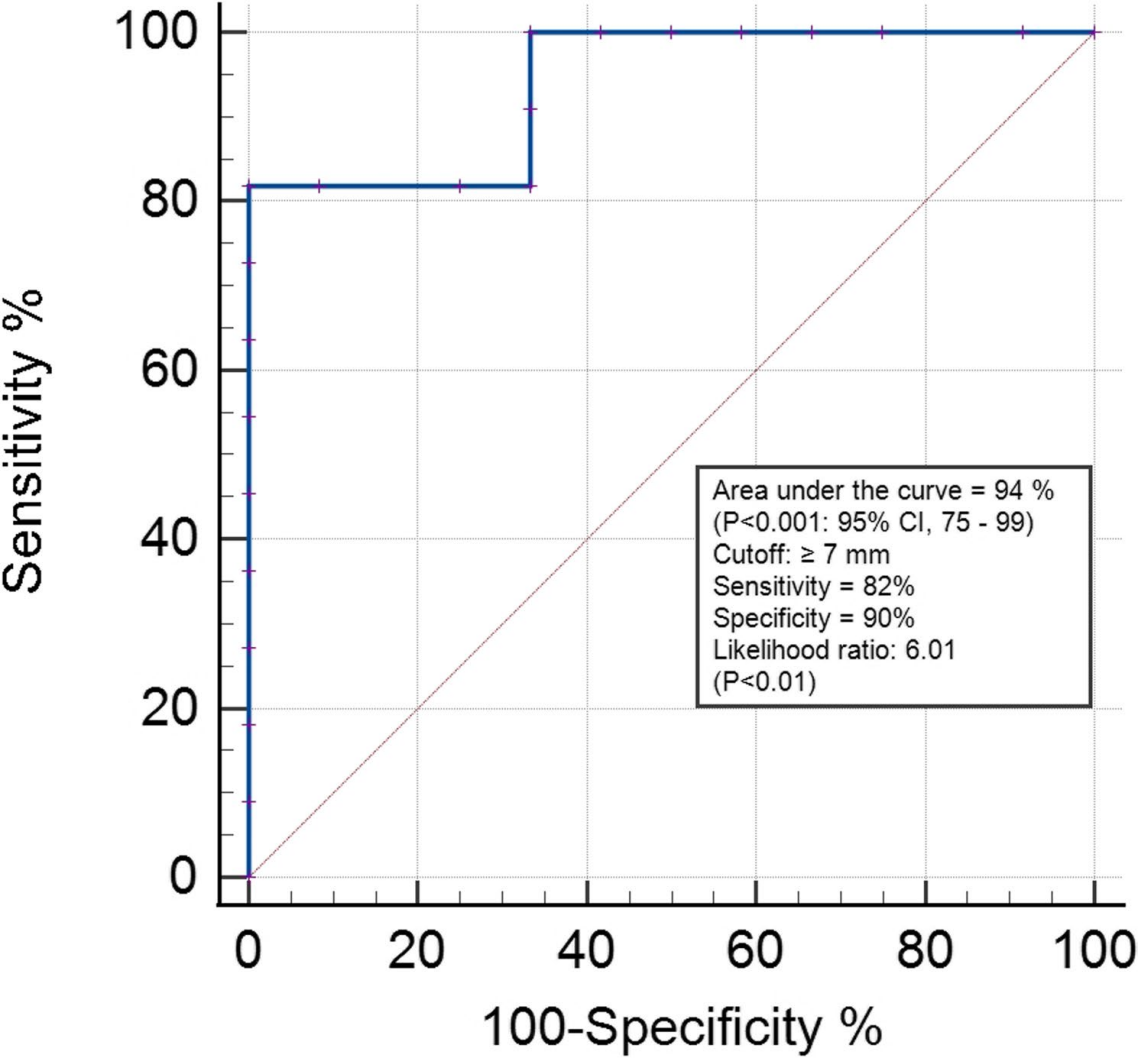
ROC curve illustrating the sensitivity, specificity, and critical threshold of ultrasound croup fat thickness for prediction of lipomobilization in donkeys (*Equus asinus*)

In addition, the ROC curve analysis showed the cutoff point ≥ 7 mm as a critical threshold for the prediction of hyperlipidemia in donkeys (*P* < 0.01). Moreover, the estimated AUC, sensitivity, specificity, and likelihood ratio were 0.94, 81%, 90%, and 6, respectively (Fig. 2).

### Effect of fasting-induced hyperlipidemia on hepatic ultrasonography

**Fig. 3:**
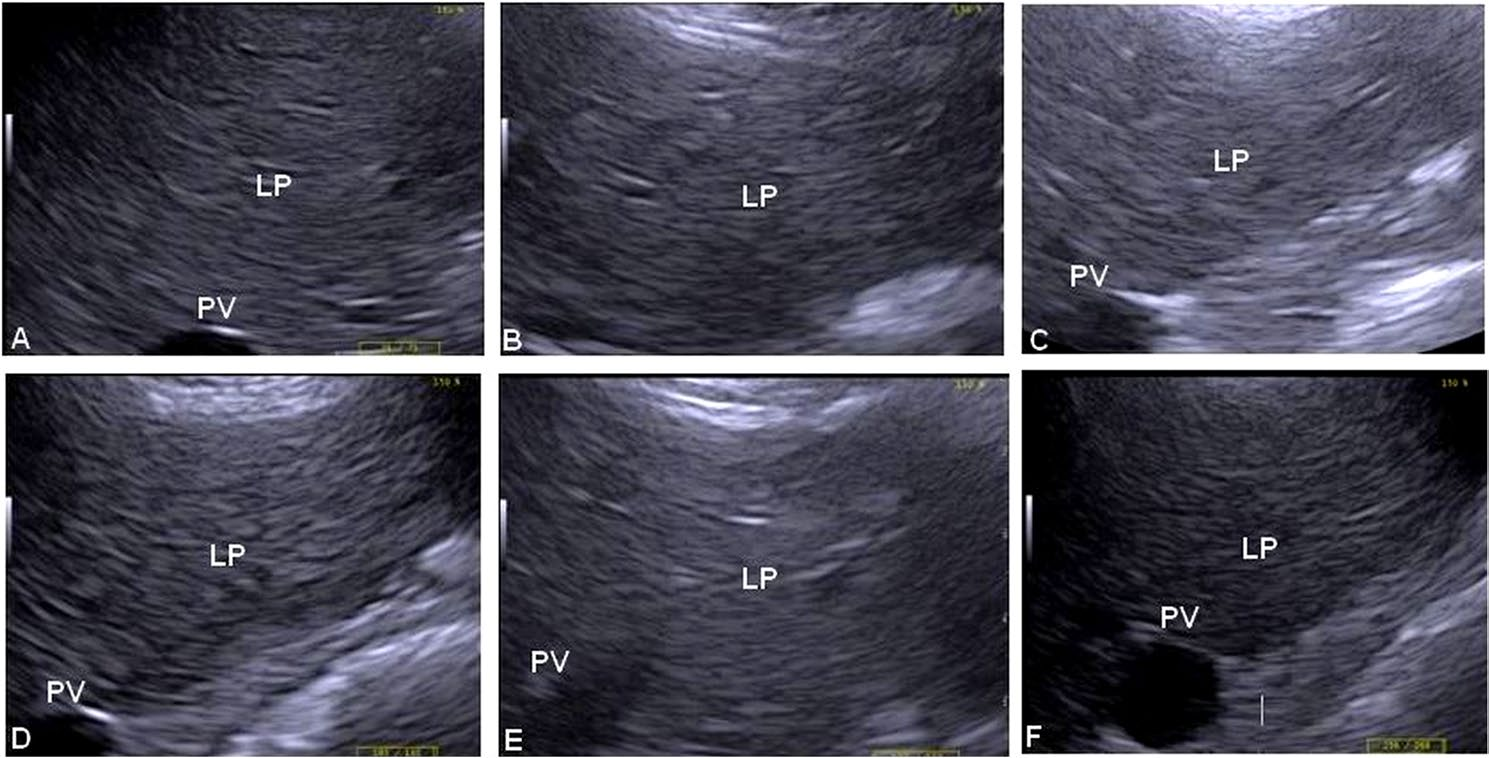
Ultrasound images of the liver, at the mid-way of the 14th intercostal space of the right side, of a donkey (fasted group) show the variations in RE during the study. Image (**A**), just before fasting, reveals 73.35 of RE. Image (**B**), after 2 days of fasting, shows 85.19 RE. Image (**C**), after 4 days of fasting, represents 108.07 RE. Image (**D**), after 5 days of the study, indicates 89.3 RE. Image (**E**), after 7 days of the study, reveals 85.53 echogenicity. Image (**F**), after 10 days of the study, shows 82.22 RE

**Fig. 4:**
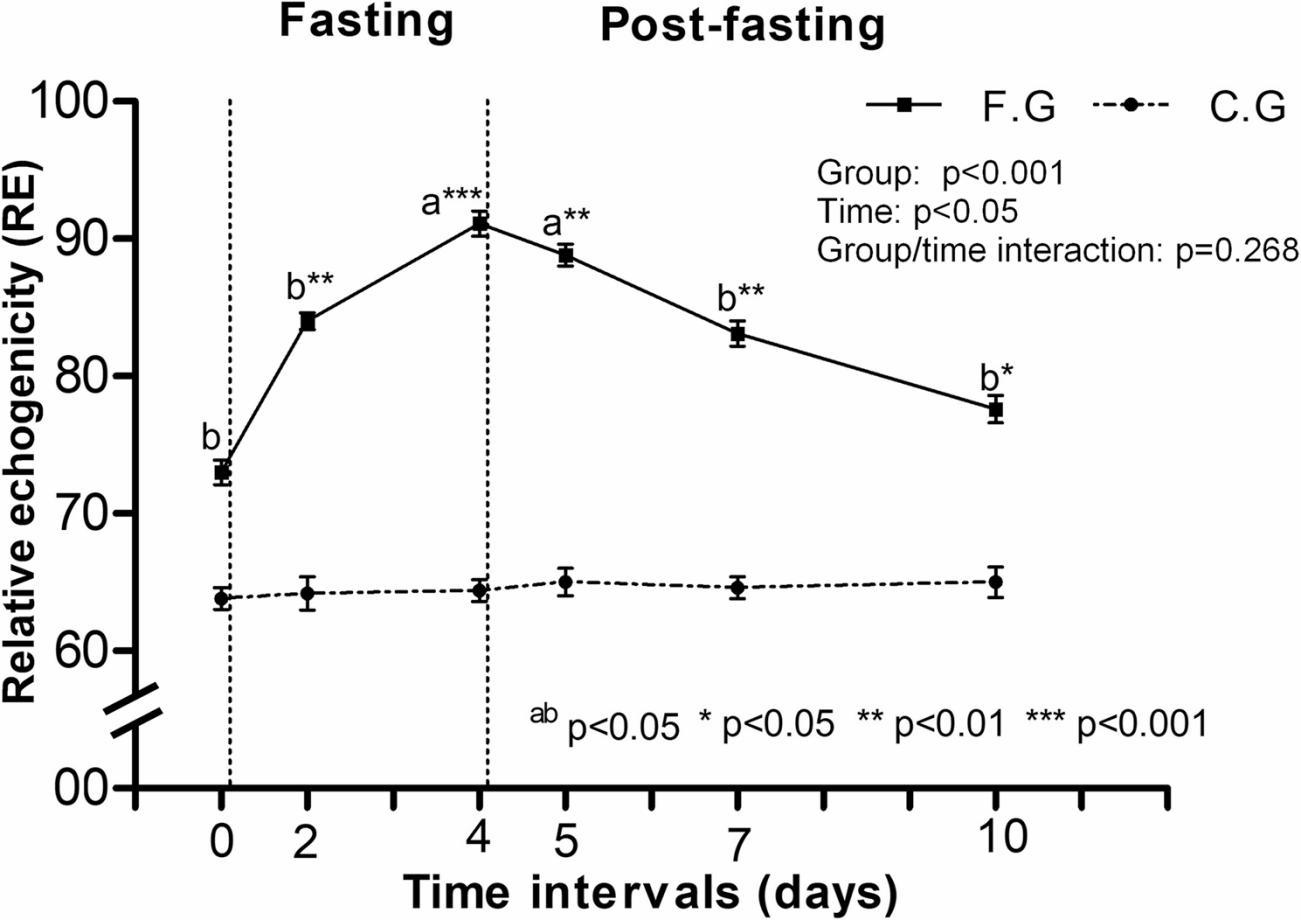
Pattern of relative echogenicity (RE) of the liver in donkeys of fasting (■) and control (●) groups throughout the study

**Fig. 5:**
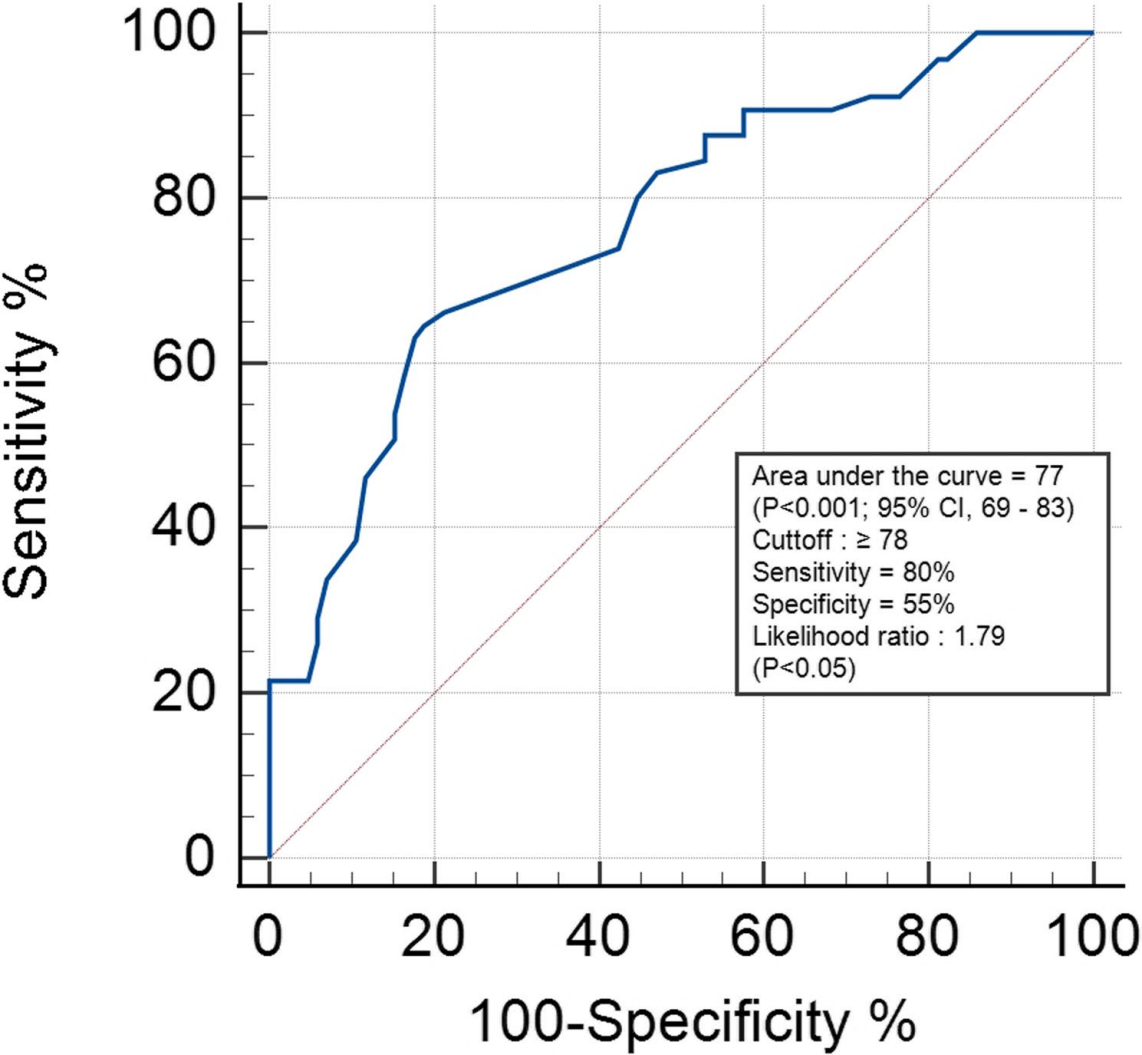
ROC curve illustrating the sensitivity, specificity, and critical threshold of RE of the liver for detecting hyperlipidemia in donkeys (*Equus africanus asinus*)

The variations in the ultrasound measurements of the liver in donkeys of the fasting and control groups are displayed in Table 2. All the parameters except the diameter of PV did not significantly differ between the two groups (*P* < 0.05). Moreover, the mean PV diameter decreased after fasting in the FG and then increased at the end of the study (*P* < 0.05). Furthermore, in comparison with those of control animals, the PV diameter in the FG was lower at 2, 4, and 5 days (*P* < 0.05). Furthermore, follow-up hepatic ultrasonography of the FG revealed that the liver parenchyma was brighter in color during the fasting stage, indicating increased RE (Fig. 3), however, this increase was slight but significant. Figure 4 shows the comparative evaluation of RE between the control and fasting groups, where a significant difference was detected (*P* < 0.001). Moreover, the duration of fasting significantly influenced RE (*P* < 0.05), which peaked at 4 days of fasting in the FG. In addition, ROC analysis showed that the cutoff point of 78 was a critical threshold for diagnosing lipomobilization in hyperlipidemic donkeys with a sensitivity and specificity of 80% and 55%, respectively, and the area under the curve was 77 (*P* < 0.001) (Fig. 5).

**Table 2:** The effect of fasting-induced hyperlipidemia on the liver ultrasonographic measurements in donkeys

Parameters	Groups (Number)	Days relative to fasting^†^						Repeat measures analysis of variance^‡^		
			Fasting stage		Post-fasting stage					
		0	2	4	5	7	10	Group	Time	Group × time
Size (cm)	FG (*n* = 20)	11.9 ± 0.8	12.1 ± 0.5	11.9 ± 0.9	12.8 ± 0.8	12.0 ± 0.8	11.9 ± 0.8	0.563	476	0.828
	CG (*n* = 5)	12.4 ± 0.5	12.2 ± 0.8	12.4 ± 0.9	12.6 ± 0.9	12.4 ± 0.4	11.9 ± 0.4			
Depth (cm)	FG (*n* = 20)	2.0 ± 0.2	2.1 ± 0.2	2.1 ± 0.3	2.1 ± 0.4	2.1 ± 0.2	2.1 ± 0.3	0.120	0.299	0.111
	CG (*n* = 5)	1.9 ± 0.7	1.9 ± 0.4	1.9 ± 0.3	2.0 ± 0.3	2.0 ± 0.4	2.1 ± 0.4			
Thickness (cm)	FG (*n* = 20)	6.7 ± 0.4	6.5 ± 0.5	6.8 ± 0.3	6.5 ± 0.5	6.5 ± 0.2	6.9 ± 0.4	0.061	0.424	0.950
	CG (*n* = 5)	6.6 ± 0.9	6.5 ± 0.8	6.6 ± 0.8	6.8 ± 0.8	6.8 ± 0.6	7.0 ± 0.8			
PVD (cm)	FG (*n* = 20)	1.9 ± 0.2^a^	1.7 ± 0.2^b*^	1.6 ± 0.3^b*^	1.5 ± 0.4^b*^	1.8 ± 0.4^a^	1.8 ± 0.4^a^	0.05	0.021	0.207
	CG (*n* = 5)	1.8 ± 0.3	1.9 ± 0.3	1.9 ± 0.4	1.7 ± 0.2	1.9 ± 0.2	1.8 ± 0.3			
CVC (cm)	FG (*n* = 20)	2.5 ± 0.4	2.3 ± 0.4	2.3 ± 0.2	2.4 ± 0.4	2.5 ± 0.3	2.5 ± 0.2	0.713	0.074	0.175
	CG (*n* = 5)	2.4 ± 0.2	2.5 ± 0.4	2.4 ± 0.2	2.4 ± 0.2	2.4 ± 0.3	2.4 ± 0.2			

*Abbreviations*: *FG* fasting group, *CG* control group, *PVD* portal vein diameter, *CVC* caudal vena cava diameter

^†^ values are mean ± standard error of the mean

^‡^ F-values of groups, times relative to fasting, and group–time interactions. ^ab^Means in the same row differ significantly (*p* < 0.05)

^*^Means in the column of the same parameter differ significantly between control and fasting groups (*p* < 0.05)

#### Effect of fasting-induced hyperlipidemia on the lipid profile

**Fig. 6:**
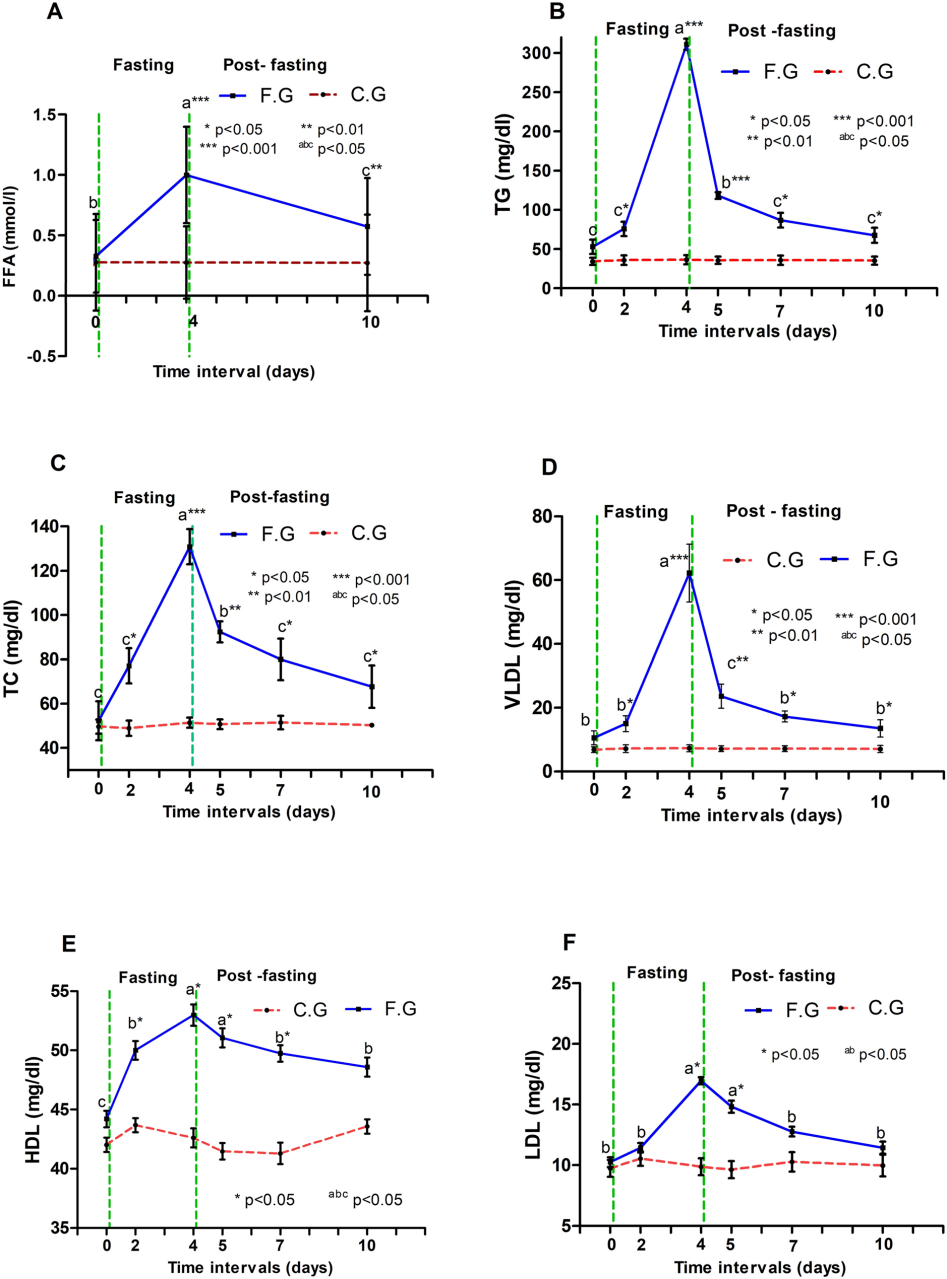
Concentrations of free fatty acids (FFA, **A**), triglycerides (TG, **B**), total cholesterol (**C**), very low density lipoprotein (VLDL, **D**) high-density lipoproteins (HDL, **E**), and low-density lipoproteins (LDL, **F**) in donkeys of fasting (■) and control (●) groups during the study period

The changes in the blood metabolites in donkeys in the control and fasting groups are shown in Fig. 6. The two groups had significant differences in the level of FFAs (*P* < 0.05). The concentration of FFAs was greater in the fasting group (*P* < 0.001). The duration of fasting influenced the mean FFA levels (*P* < 0.001). Moreover, repeated-measures analysis of variance indicated significant group/time interactions for FFAs (*P* < 0.001). Figure 6 illustrates the changes in the lipid profile of donkeys throughout the study, where the serum concentrations of TG, total cholesterol, VLDL, HDL, and LDL were significantly different between the fasting and control groups (*P* < 0.001). 4 weeks of fasting, the mean TG, total cholesterol, and VLDL were significantly greater in the fasting group than in the control group (*P* < 0.001). Compared to those in control donkeys, the levels of HDL and LDL were significantly greater in the fasted animals after 4 days of fasting (*P* < 0.05). The duration of fasting had significant effects on the mean TG (*P* < 0.001), total cholesterol (*P* < 0.001), VLDL (*P* < 0.001), HDL (*P* < 0.05), and LDL (*P* < 0.001) levels, as their concentrations peaked at 4 days of fasting in the fasting group (*P* < 0.05) and then decreased significantly after fasting, reaching approximately the baseline levels at 10 days of study. Furthermore, significant group/time interactions were detected for TG (*P* < 0.001), total cholesterol (*P* < 0.001), VLDL (*P* < 0.001), HDL (*P* < 0.01), and LDL (*P* < 0.001), indicating that their values differed between groups over time.

##### The relationships among lipid profile indices and ultrasound measurements of CFT and LE

**Fig. 7:**
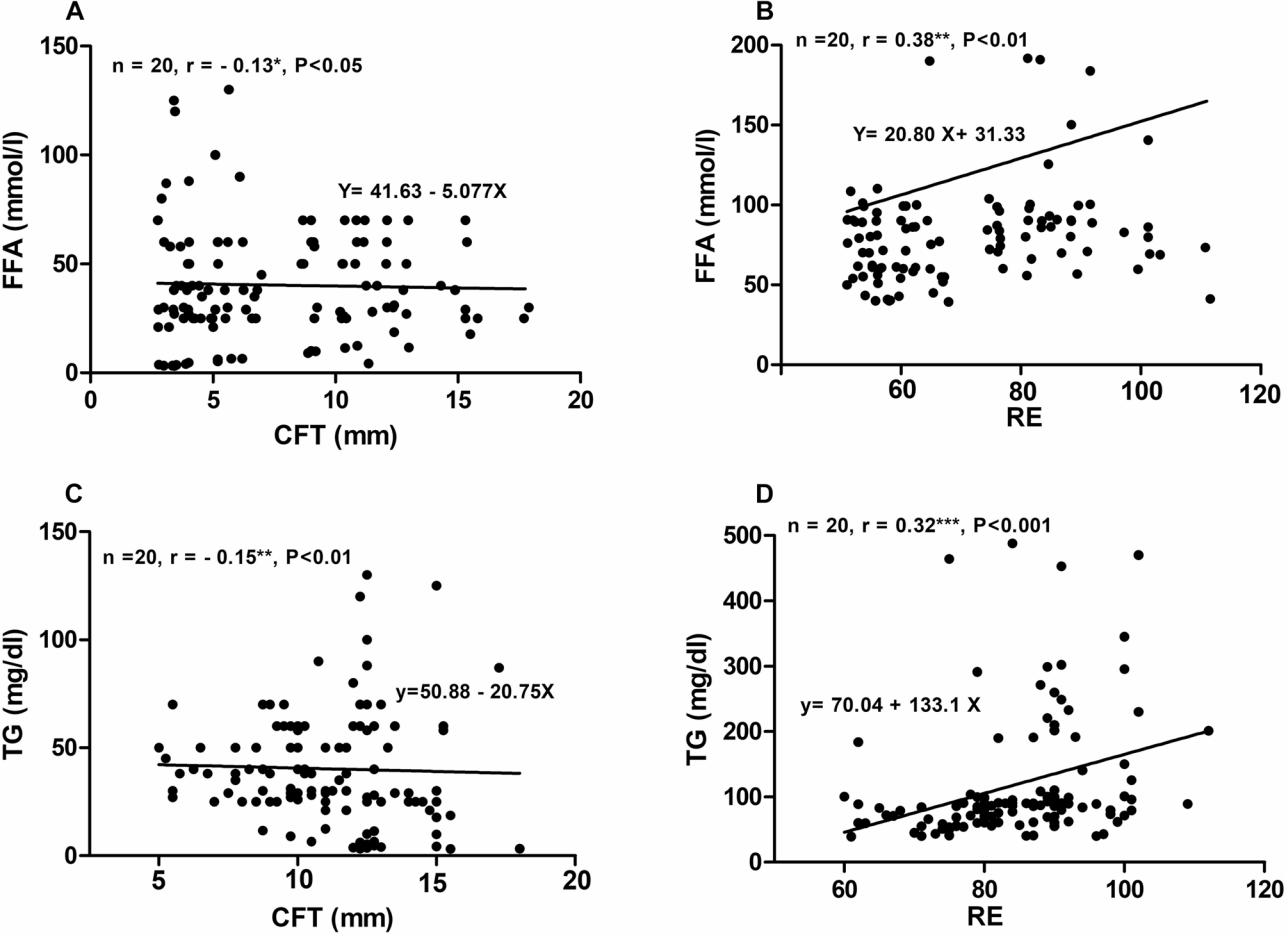
Scatter plots for the relationships between free fatty acids (FFA) and ultrasound croup fat thickness (CFT) (**A**), FFA and relative echogenicity of the liver (RE) (**B**), triglycerides (TG) and CFT (**C**), and TG and RE (**D**)

The association between lipid profile parameters and ultrasound measurements of CFT and RE is summarized in Table 3. A weak negative correlation coefficient (*r*=−0.13, *P* < 0.05) was determined between FFA and CFT (Fig. 7A). In contrast, a positive moderate correlation coefficient (*r* = 0.38, *P* < 0.01) was obtained between FFA and RE (Fig. 7B). Moreover, a weak correlation was observed between TG and CFT (*r*=−0.12, *P* < 0.05) (Fig. 7C), and a moderate relationship was determined between TG and RE (*r* = 0.32, *P* < 0.01) (Fig. 7D).

**Table 3:** Pearson correlation coefficients among croup fat thickness (CFT), liver echogenicity (LE), and lipid profile indices:

	TG	TC	LDL	HDL	VLDL	CFT	RE	FFA
Triglyceride (TG)	1							
Total cholesterol (TC)	0.91**	1						
Low-density lipoproteins (LDL)	0.66**	0.80**	1					
High-density lipoproteins (HDL)	0.50**	0.42**	0.41**	1				
Very low-density lipoproteins (VLDL)	0.61**	0.31**	0.22*	0.16*	1			
Croup fat thickness (CFT)	−0.15*	0.16	−0.21	−0.45*	−0.36*	1		
Relative echogenicity (RE)	0.32**	0.44**	0.55**	0.12	0.22	−0.22	1	
Free fatty acids (FFA)	0.81**	0.71**	0.67**	0.33**	0.36**	−0.13*	0.38**	1

*P < 0.05, ** *p* < 0.01

## Discussion

Severe systemic and gastrointestinal diseases are typically associated with variable degrees of reduced feed intake [14–16], resulting in subsequent lipids, carbohydrates, and proteins, which make a large impact on energy metabolism [2, 9, 17]. To the best of the authors’ knowledge, no reports are available describing the effect of fasting-induced hyperlipidemia on ultrasound measurements of CFTs and GMTs or hepatic ultrasonographic echogenicity in donkeys. Therefore, this study was designed to investigate the utility of ultrasound CFT and RE as indicators for lipomobilization associated with fasting-induced hyperlipidemia in donkeys.

In this study, despite the subjectivity of BCS and BW in evaluating body condition, they were included to determine their usefulness in estimating lipomobilization. The mean values of BW and BCS did not significantly differ between the FG and baseline values. This result could be explained by the fact that both techniques are subjective tools for estimating body condition, as BW was calculated mathematically using the formula reported by Pearson and Quassat [18], and BCS was evaluated via inspection and palpation [19]. Using a mathematical Eq. [6], the actual body weight was underestimated by an average of 7 kg and an average error of 4.5% in Hucul horses [20]. The BCS was not a useful index for detecting early weight loss as a result of dietary restriction in pony mares [3]. In contrast, Brinkmann et al. [21] reported that the BCS is a good indicator of rapid health problems caused by undernourishment in horses when kept under seminatural conditions for the long term. These differences may be due to the use of different study designs.

In the present study, ultrasound CFTs decreased significantly during fasting, indicating that lipid mobilization is utilized as a source of energy due to a negative energy balance. In equines, a negative energy balance may lead to an increased rate of lipolysis [2, 17]. As an objective procedure, ultrasound CFT may be a reliable technique for evaluating body condition in donkeys. Ultrasound measurements of CFTs were established as a screening technique for monitoring body condition in donkeys [8], dairy cattle [22], and buffaloes [23]. Dugdale et al. [3] reported decreased subcutaneous fat depth on ultrasound in pony mares exposed to feed restriction.

The ROC curves were used to determine the critical thresholds of ultrasound measurement of CFT as a detector for hyperlipidemia in donkeys. Donkeys with ultrasound CFT ≥ 7 mm were approximately 6 times more likely to have hyperlipidemia than animals below this threshold. This value of CFT can be used as a predictor for fasting-induced hyperlipidemia. Furthermore, the present result was supported in previous studies [9, 12], in which the authors concluded that the prevalence of hyperlipidemia in fat and obese equines is higher than in normal and thin animals, which could be attributed to high body fat reserves in overconditioned donkeys [2].

Moreover, ultrasound GMT did not change during fasting, suggesting that the fasting stage was not drastic enough to break down muscular protein. Decreased feed intake in equines is usually accompanied by hyperlipidemia with subsequent fat mobilization, and protein breakdown, which are used as energy sources [2, 9].

As a noninvasive tool, ultrasonography is a commonly used technique in donkeys [11, 24]. The present research revealed increased RE during fasting, which could be attributed to hepatic fatty infiltration. With poor-quality roughages, donkeys survive harsh environmental conditions well, increasing susceptibility to pathological conditions associated with a negative energy balance such as excessive lipolysis with subsequent hepatic lipidosis [25].

In the present study, donkeys with RE of ≥ 78 were 1.8 times more likely to have hyperlipidemia than animals with RE below this critical threshold. Despite moderate sensitivity (80%) and specificity (55%) were determined, the area under the curve (AUC = 77) was very accurate. This finding may be useful for the diagnosis or confirmation of liver fatty infiltration that accompanies hyperlipidemia. In previous studies [2, 9], the authors stated that excessive fat mobilization may overwhelm the capacity of the liver to process triglycerides into VLDL, leading to triglyceride accumulation with subsequent development of hepatic lipidosis. According to the authors’ knowledge, no reports have previously discussed the relationship between RE and hepatic lipidosis. However, RE was positively correlated with fatty liver in rats [26].

In the present study, a decreased PV diameter was observed during fasting, possibly due to either decreased portal blood flow as a result of fasting or as a complication of fatty infiltration with subsequent constriction of the vein. In a previous human study [27], the authors reported increased portal blood flow velocity in patients with liver cirrhosis post-meal. Moreover, reduced portal blood flow was observed in mice with fatty liver, which was explained by increased intrahepatic pressure [28]. In this work, other hepatic ultrasound measurements, including size, depth, and thickness, were unchanged in the FG, indicating no hepatomegaly despite fatty infiltration, which may be of a mild degree. As reported elsewhere [29], liver parenchymal swellings are particularly pronounced when the hepatic triglyceride content exceeds 150 mg/g of liver fresh weight. As a limitation of the present study, liver TG concentrations were not determined to avoid the complications and stress of the liver biopsy technique [11], especially during fasting stage.

In the present research, lipid profile biomarkers, including TG, TC, VLDL, HDL, and LDL, were significantly increased during fasting, indicating development of hyperlipidemia as a result of lipid mobilization. The development of a negative energy balance may induce inappropriate and excessive mobilization of peripheral fat stores [9, 29], with subsequent release of free fatty acids, which are converted into triglycerides within the liver, and the exported VLDLs accumulate within the circulation [30]. As expected, the donkeys in this study uniformly responded to fasting; their serum lipid indices increased, and hyperlipidemia was developed by previous studies [1, 12, 31]. Mobilized fats may be utilized directly by muscle or transported to the liver, where they are completely oxidized by the tricarboxylic acid cycle (TCA), exported as lipoproteins and TG, or partially oxidized to ketone bodies [2]. The pathway for ketone body formation is not well understood in equines and is unlike ruminants; therefore, TG increases in the liver with subsequent increased formation of VLDL [9, 17, 30]; thus, the level of beta-hydroxybutyrate, as a ketone body, was not measured in the present study. The biochemical etiology of hyperlipidemia in this study may be due to the overproduction of TG rather than the failure of TG catabolism. As mentioned in previous reports [2, 9, 21, 31], if the liver’s ability to process delivered fatty acids is exceeded, equines release large amounts of mobilized lipids back into the bloodstream.

In the current study, the increased serum concentrations of FFAs in the FG could be explained by the effect of a negative energy balance and subsequent mobilization of fat stores. As stated in a previous report [32], hormone-sensitive lipase activity increases during fasting, resulting in the net release of FFAs into the bloodstream. The increase in FFA levels in the present work was in accordance with the findings of previous studies in horses [1, 33–35]. It was reported that the activity of hormone-sensitive lipase increases during fasting, resulting in increased blood levels of FFAs [3]. Furthermore, the concentrations of FFAs may be varied in donkeys that were supplemented with different energy sources [36], and those were exposed to different management systems [37].

In this research, low negative correlations were observed between CFT and FFA and CFT and TG. The observed low relationships may be due to the mobilized fats are usually released not only from croup fat, but also form other body fat stores. In equine, if a negative energy balance, several signals interact to maintain normoglycemia, resulting in further energy release from the different peripheral body fat reserves [9]. In a previous study in donkeys [7], the author listed six anatomical sites for ultrasound measurements of subcutaneous peripheral body fat stores. Moreover, moderate positive correlations were noticed between RE and FFA and RE and TG in this study, indicating the presence of individual variations in the degrees of lipomobilization and/or hepatic fatty infiltration. Hughes [2] reported that variable extents of fat mobilization and hepatic lipidosis could be observed in equines with hyperlipidemia.

In the present study, most of the observed variations related to ultrasound CFT, RE, and serum lipid profile indices improved post-fasting, indicating most of the tested variables were reversible. This may be explained by the short-term effect of the hyperlipidemic stage, suggesting the therapeutic intervention was unnecessary, and adequate refeeding, as reported in this study, might be sufficient. This suggestion should be taken with caution, especially in severely hyperlipidemic donkeys. In a recent study [38], the authors recommended rehydration therapy with Ringer’s lactate alongside nutritional management in a hyperlipaemic Jenny. In contrast, it has been reported that insulin, heparin, and glucose injections are indicated during the therapeutic protocol of hyperlipidemia in donkeys [25].

### Limitations of the study

The small size of animals in the control group and the lack of histopathological confirmation of hepatic lipidosis are limitations of the present study. As a prospective controlled study, the control group was not tested for the fasting effect; therefore, the number of animals in this group was somewhat small. The histopathological examination was not conducted in this research to avoid the stress and complications that may develop in fasted animals as a result of the liver biopsy technique. However, despite the previous limitations, the present study is a reasonable starting point for monitoring hyperlipidemia in donkeys.

## Conclusion

Reduction of the subcutaneous fat thickness may be developed in donkeys with fasting-induced hyperlipidemia. Moreover, the ultrasound measurement of CFT is a useful predictor for lipolysis, where donkeys with a critical threshold of ≥ 7 mm are 6 times more likely to develop hyperlipidemia post-fasting, confirming that obesity is a risk factor for hyperlipidemia in donkeys. Furthermore, increased RE suggests hepatic lipidosis may develop in donkeys as a complication of fasting; therefore, a critical threshold of ≥ 78 can be used as a diagnostic tool for this disease. Moreover, the alterations in the lipid metabolites as a result of fasting are usually reversible post-fasting; therefore, the treatment may be unnecessary especially in less severe cases.

## Methods

### Animals and study design

The Animal Care and Welfare Committee of the Faculty of Veterinary Medicine, Assiut University, Assiut, Egypt, ethically approved this research (06/2024/0261). All national and institutional guidelines for the care and use of animals in experimentation were followed during the study procedures, and all animals were housed and cared for according to the Egyptian Animal Welfare Act (No. 53, 1966). This study was performed in compliance with the ARRIVE guidelines and regulations. All rules and procedures for animal care and use were followed throughout the study.

The present research was conducted on 25 clinically healthy adult donkeys (*Equus africanus asinus*) (9 males and 16 non-pregnant and non-lactating females) of both sexes from a private stud in Assiut governorate (Latitude: 27.1860° N, and Longitude: 31.1680° E), where the relative humidity ranged from 26 to 35%. The average age was 4.5 ± 0.8 years (mean ± SD). The parity of female animals ranged from 1 to 3. Both groups were housed separately in two identical paddocks (6 × 20 m) with an open outdoor shelter. The space area for each donkey was 3.5m^2^. The paddocks were bedded on wood shavings. The donkeys underwent physical and hematological examinations before enrolling in the study. Before the start of this research, all animals were examined for parasitic infections; no internal parasitic infections were observed. However, all the donkeys were dewormed using piperazine citrate (Stavro International Co, Egypt) orally (dose, 100 mg/kg body weight). Two weeks later, the dose was repeated.

**Fig. 8:**
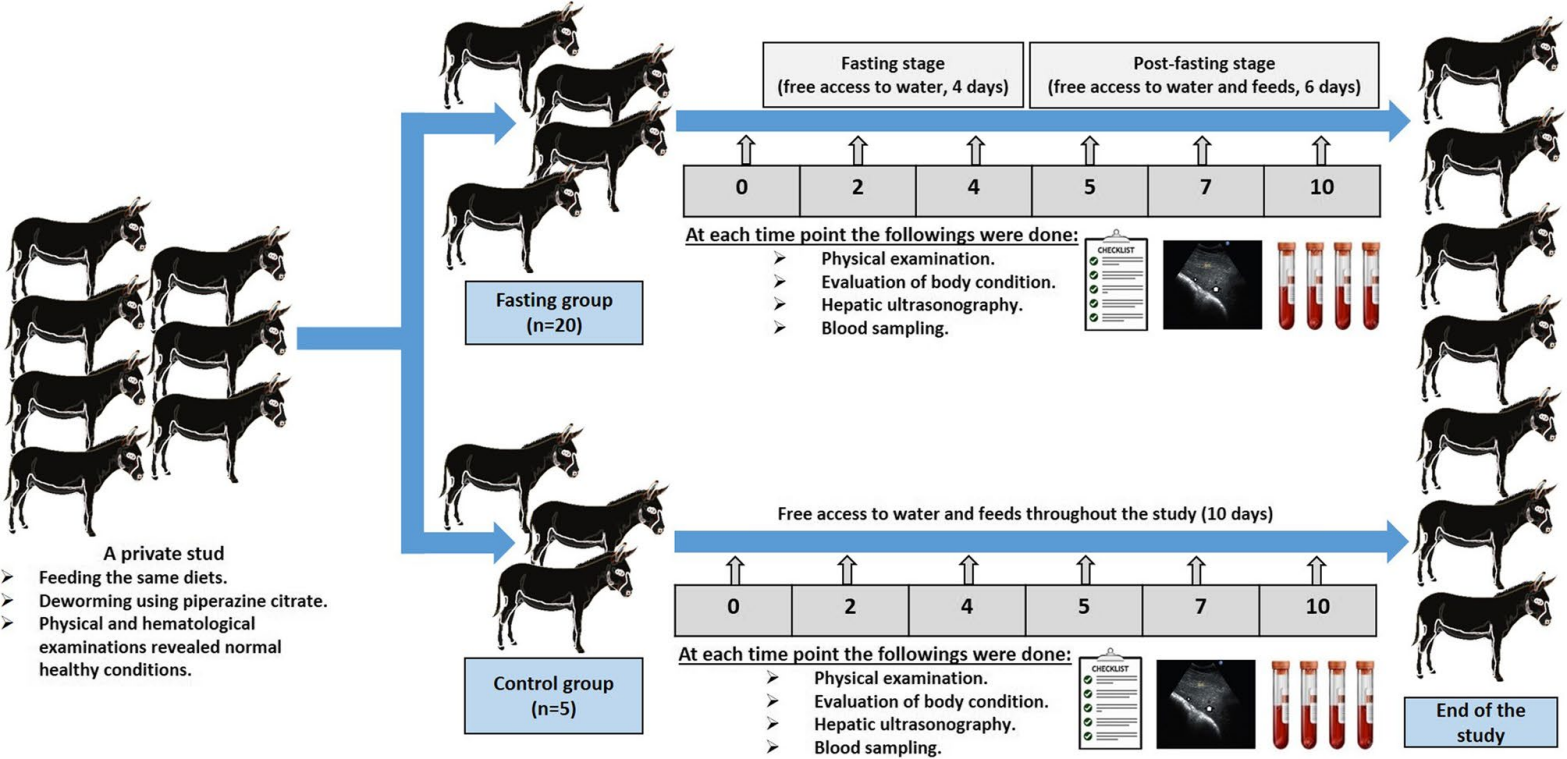
Schematic representation of the study design

Before the experiment, the feed was distributed in a way that minimized competition among donkeys. Any uneaten food after each feeding was removed to prevent spoilage and maintain a clean feeding area. Moreover, providing a calm and quiet environment during feeding times to reduce stress and encourage consumption was ensured. A fresh and clean water source was available. Initially, all the donkeys were fed green fodder in the form of Egyptian clovers (Barseem, *Trifolium alexandrinum*), and the concentrate mixtures consisted of chopped wheat straw and maize provided with a mineral mixture. The present study was conducted as a prospective observational control longitudinal study, in which the animals were classified randomly into two groups: fasting (FG, *n* = 20) and control (CG, *n* = 5) groups. In the FG, the experiment period (10 days) consisted of two stages: the fasting stage (4 days) and the post-fasting stage (6 days). The CG had free access to water and feeds throughout the experimental period, while the FG had free access to water only during the fasting stage (Fig. 8). During the post-fasting stage, the donkeys in the FG were monitored closely for an additional 6 days and gradually refed with small quantities of Egyptian clovers every 4 h for 24 h [13], to avoid any digestive upset or any potential health complications, and then offered *ad libitum* clovers and concentrates like the control group.

### Assessment of body condition

Throughout the study, using visual inspection and palpation, the body condition score (BCS) for all donkeys was evaluated using a 1–5 scale according to the chart previously reported by Burden [19]. The donkeys’ body weight (BW) was calculated as described by Pearson and Quassat 1996 [18]:

$$\begin{aligned}\mathrm{Body}\;\mathrm{weight}\;(\mathrm{kg})=&(\mathrm{heart}\;\mathrm{circumference}\;{\lbrack\mathrm{cm}\rbrack}^{\rbrack2.12})\\&\times(\mathrm{length}\;{\lbrack\mathrm{cm}\rbrack}^{0.688})/380\end{aligned}$$

The heart girth is the circumference measured by a measuring tape from the caudal edge of the withers around the girth behind the elbow, and the length is the distance from the olecranon process of the elbow to the tuber ischii.

The subcutaneous croup fat thickness (CFT), subcutaneous fat depth overlying the gluteal region) and gluteal muscle (GMT) thicknesses were measured using a multifrequency linear probe (6–10 MHz, MyLabOne VET, Esaote, Italy), where the probe was placed on the flat area between the tuber coxae and tuber ischii anterior to the tail-head and parallel to the midline [8]. Formerly, this area was shaved, washed, and disinfected with 70% alcohol, after which the gel was applied. After freezing the ultrasound images, the distance from the skin to the fascia covering the gluteal muscle was defined as the CFT. While the distance from the profound fascia to the hyperechoic line formed by the pelvic bone was taken as the GMT.

### Hepatic ultrasonography

Hepatic ultrasound was carried out on the standing posture using a real-time B-mode scanner with a multifrequency microconvex transducer (MyLabOne VET, Esaote, Italy) [11]. The frequency was adjusted at 5 MHz. First, for the preparation of donkeys for hepatic ultrasonographic examination, the area from the 7th to 15th intercostal space at the right side was clipped and shaved, and a coupling gel was applied. Hepatic ultrasonographic imaging was performed by placing the probe in each intercostal space and then moving it ventrally parallel to the ribs. The dimensions of the liver, including size, depth, and thickness, were measured. The hepatic size was calculated as the difference between the distances between the dorsal and ventral margins of the liver. Moreover, if visible, the location and diameter of the caudal vena cava and portal vein were measured. Furthermore, hepatic ultrasonographic images were stored for subsequent offline analysis to determine the RE. Stored images were converted from 8-bit RGB images to 8-bit grayscale images, subsequently, the images were digitized, and the mean echogenicity (ME) of the region of interest (ROI) (defined as the mean pixel brightness or mean gray level of the liver) was calculated using image processing software (ImageJ, National Institutes of Health, Bethesda, Maryland, USA) with a scale of 255 Gy levels (0 = black; 255 = white). The gray distribution appears as a histogram, and the software system automatically calculates the mean value (echogenicity). Values of probe frequency, gain, depth, and place at the same intercostal space on the animal were kept the same throughout the study for accurate measurements of RE.

### Blood sampling and laboratory analysis

Blood samples from each donkey, one with sodium fluoride and the other without an anticoagulant, were collected by jugular venipuncture. Blood samples (baseline, T0) were collected from both groups of donkeys approximately 4 h before the morning feeding. Further blood samples were taken from the fasting group after 2 and 4 days of fasting and after 5, 7, and 10 days of study. In the control group, blood samples were drawn at 2, 4, 5, 7, and 10 days. The time of blood sampling was set to be at 10:00. Blood samples were centrifuged, and serum and plasma samples were harvested and stored in Eppendorf tubes at − 20 °C until analysis. The serum samples were assayed for the levels of insulin, free fatty acids, triglycerides, total cholesterol, high-density lipoprotein (HDL), and low-density lipoproteins (LDL). The plasma samples were assayed for glucose levels. The concentrations of FFAs and insulin were measured using commercial ELISA kits supplied by Sunlong (Sunlong Biotech Co., Ltd, China). The biochemical indices including TG, TC, HDL, LDL were determined using commercial test kits according to the instructions of the manufacturer (Spectrum Diagnostics, Egypt), and UV spectrophotometer (Optizen 3220 UV, Mecasys Co, Ltd, Korea) was used for this purpose.

### Statistical analysis

The data are presented as the means ± standard errors of the means (SEs). For statistical evaluation, the data were analyzed using IBM SPSS Statistics (Version 25, Munich, Germany). First, the normality of all the variables was tested using the Kolmogorov-Smirnov test. All the parameters were normally distributed. Moreover, a repeated measures analysis of variance was calculated using the linear mixed procedure, where the donkeys’ numbers were the subjects and the time relative to sampling was set as a repeated variable. The model included terms for time relative to sampling, donkey groups, and time × group interaction. The following linear mixed model was used for analyzing the effects of time, donkey groups, and their interactions:

$$Mjk = \mu + Tj +Gk + TGjk +\varepsilon jk$$

where *M* = the observed level of the tested parameter, *µ* = the overall mean, *T*_*j*_ = the fixed effect of time (*j* = 0, 2-day, 4-day, 5-day, 7-day, 10-day), *G*_*k*_ = the fixed effect of the donkey groups (*k* = 1, 2), *TG*_*jk*_ = Timej and Groupk interaction, and *εjk* = residual error. The variations within the same group at different time points were assessed using the least significant difference (LSD), and a one-way analysis of variance was used for comparison between FG and CG. The receiver operating characteristic (ROC) analysis was used to determine the cutoff points and critical thresholds of the investigated variables. The ROC curves were used to test sensitivity% versus 100 − specificity%. The sensitivity was the proportion of donkeys diagnosed with disease at or above a given threshold. Moreover, the specificity was the proportion of animals that did not have disease within a given threshold [39]. The point on the ROC curve with the highest combined sensitivity and specificity was considered the critical threshold. Furthermore, this crucial threshold was interpreted based on the area under the curve (AUC) such that if the AUC = 50, it was noninformative; if 50 < AUC ≤ 70, it was accurate; if 70 < AUC ≤ 90, it was very accurate; if 90 < AUC < 100, it was highly accurate; and if AUC = 100, then it was considered perfect [40].

## Data Availability

The datasets generated and/or analyzed during the current study are available from the corresponding author on reasonable request.

## Abbreviations

BCS: Body condition score
FFA: Free fatty acids
FG: Fasting group
CG: Control group
CFT: Croup fat thickness
CVC: Caudal vena cava
HDL: High-density lipoprotein
LDL: Low-density lipoprotein
PV: Portal vein
RE: Relative echogenicity
ROC: Receiver operating characteristics
TCA: Tricarboxylic acid cycle
TG: Triglycerides
TC: Total cholesterol
VLDL: Very low-density lipoprotein
